# PIF-Mediated Sucrose Regulation of the Circadian Oscillator is Light Quality and Temperature Dependent

**DOI:** 10.3390/genes9120628

**Published:** 2018-12-13

**Authors:** Ekaterina Shor, Raya Potavskaya, Ayelet Kurtz, Inyup Paik, Enamul Huq, Rachel Green

**Affiliations:** 1 Department of Plant and Environmental Sciences, Institute for Life Sciences, Edmond J. Safra Campus, Givat Ram, Hebrew University, Jerusalem, 91904 Israel; ekaterin.shor@mail.huji.ac.il (E.S.); poltavskaya.raya@gmail.com (R.P.); ayelet.kurtz@mail.huji.ac.il (A.K.); 2 Department of Molecular Biosciences and The Institute for Cellular and Molecular Biology, The University of Texas at Austin, Austin, TX 78712, USA; inyuppaik@gmail.com (I.P.); huq@austin.utexas.edu (E.H.)

**Keywords:** circadian rhythms, sucrose signal, PIFs, red/blue ratio, temperature

## Abstract

Studies are increasingly showing that metabolic and circadian (~24 h) pathways are strongly interconnected, with the circadian system regulating the metabolic state of the cell, and metabolic products feeding back to entrain the oscillator. In plants, probably the most significant impact of the circadian system on metabolism is in its reciprocal regulation of photosynthesis; however, the pathways by which this occurs are still poorly understood. We have previously shown that members of the basic helix-loop-helix (bHLH) transcription factor PHYTOCHROME INTERACTING FACTOR (PIF) family are involved in the photosynthate entrainment of the circadian oscillator. In this paper, using *Arabidopsis* mutants and overexpression lines, we examine how temperature and light quality affect PIF-mediated sucrose signaling to the oscillator and examine the contributions of individual PIF members. Our results also show that the quality of light is important for PIF signaling, with red and blue lights having the opposite effects, and that temperature affects PIF-mediated sucrose signaling. We propose the light sensitivity of PIF-mediated sucrose entrainment of the oscillator may be important in enabling plants to distinguish between sucrose produced de novo from photosynthesis during the day and the sucrose products of starch degradation at the end of the night.

## 1. Introduction

Circadian, ~24 h endogenous time-keeping systems, enable plants to anticipate and adapt to day/night and seasonal changes in their environment. At the core of the system is the oscillator that generates the rhythms. In *Arabidopsis*, the circadian oscillator consists of a series of interlocking feedback loops, in which components regulate each other on different levels. A central loop comprises transcription factors that regulate each other’s expression. Products of *CIRCADIAN CLOCK ASSOCIATED 1* (*CCA1*) and *LATE ELONGATED HYPOCOTYL* (*LHY*), whose expression peaks at dawn, affect the expression of daytime-active genes *PSEUDO-RESPONSE REGULATORs* (*PRR*s), evening-phased genes—(*TOC1, GIGANTEA* (*GI*), and elements of the evening complex (EC): *LUX ARRHYTHMO* (*LUX*) and *EARLY FLOWERING 3/4* (*ELF3/4*). Expression of *CCA1* and *LHY* is, in turn, repressed by TOC1 and PRRs) [1,2,3]. PRRs also repress the expression of *REVEILLE8 (RVE8)* [4]. Other members of the REVEILLE family of transcription factors are, partially redundantly, involved in control of the clock pace [5]. Post-transcriptionally, the oscillator is regulated by alternative splicing and the control of mRNA stability [6,7,8]. On post-translational level, oscillator function is shown to be regulated by a time-dependent control of PRR5 and TOC1 protein degradation [9,10]. Among numerous biological processes regulated by the circadian oscillator in plants are scent production, defense responses, photosynthetic capacity and hormone production, and the control of photoperiodic responses such as flowering [3,11]. Moreover, even though circadian oscillators are extremely robust, they are adjustable and entrained by the environment. 

In the natural world, both light and temperature change throughout the day: for example, cooler nights and warmer days, or low levels of blue-enriched light just before sunrise, compared to red-enriched light during the day. Both temperature and light affect the oscillator. Not surprisingly, given the importance of accurate circadian timing, the plant circadian system is extremely sensitive to light quality and quantity, either perceived directly, via photoreceptors, or indirectly, via photosynthetic products, the most important one being sucrose. Such multi-faceted entrainment may ensure that photosynthesis, one of the most important processes regulated by the circadian oscillator, is optimized at the same time as other circadian-controlled processes are correctly regulated [3,12]. 

Several recent studies have focused on understanding how photosynthates entrain the oscillator. The oscillator components PRR7 and GI have been shown to be involved in distinct sucrose signaling pathways [13,14]. In the dark, metabolizable sugars stabilize GI protein. During the day, repression of *PRR7* modulates *CCA1* expression to adjust the phase of the oscillator; exogenous sucrose shortens the circadian period in wild type but not in *prr7-11* or *cca1-11* mutants. By contrast, in the dark, mutations CCA1 and PRR7 do not affect sucrose responsiveness of the oscillator. A bZIP family of transcription factor called bZIP63 also directly controls the expression of *PRR7* and mediates sucrose signaling to the oscillator [15]. In previous work, we established that the photosynthate entrainment of the oscillator is also mediated by members of the bHLH transcription factor PHYTOCHROME INTERACTING FACTOR (PIF) family [16,17]. In Arabidopsis, the PIF family comprises seven members (PIF1, PIF3–8), and we have shown that a higher order, quadruple (*pif1*, *pif3*, *pif4* and *pif5*) mutant (*pifQ*) has longer period rhythms in response to exogenous sucrose in a range of red light intensities from 1–75 μmol m^−2^s^−1^ as well as in white light and in the dark. Our results also demonstrated that PIFs show sucrose-dependent binding to the promoters of *CCA1* and *LHY*, but not *PRR7*, at subjective dawn [16] and we suggested that this binding directly affects circadian timing. 

In this paper, we further explore how the PIF-mediated sucrose entrainment of the oscillator is dependent on light quality and the roles of individual PIFs. Many studies have shown that PIFs can act as hubs in signaling pathways from diverse environmental signals including changes in temperature. For example, flowering time acceleration by increasing temperature is mediated by PIF4 [18] and we show how temperature affects PIF-regulated sucrose entrainment of the oscillator. 

## 2. Materials and Methods

### 2.1. Plant Materials and Growth Conditions

The Arabidopsis (*Arabidopsis thaliana*) ecotype Columbia-0 (Col-0) was used as the background for all the experimental lines. *pifQ* (*pif1pif3pif4pif5*), *pifQ CCA1:LUCIFERASE* (*LUC*) and wild-type (wt) *CCA1:LUC* transgenic plants are described in [16,19]. The *PIF*- overexpression (*PIF-ox*) lines used were *35spro:PIF1-HA* [20], *35spro:PIF3-myc* [21], *35spro:PIF4-myc* and *35spro:PIF5-myc* [22]. For all experiments, seeds were imbibed and cold-treated at 4 °C for 4 days and sown onto Petri dishes with Murashige and Skoog (MS) medium (Duchefa Biochemie, Netherlands) with or without 3% (*w*/*v*) sucrose (for luciferase assay), or 2% sucrose (*w*/*v*) (for leaf movement assays and RT–PCR). Unless otherwise stated, plants were grown in 14 h light: 10 h dark (14 L:10 D) with 100 μmol m^−2^ s^−1^ white light supplied by Philips fluorescent lights TLD 18W/840 at 23 °C.

### 2.2. Bioluminescence Assays

For the circadian bioluminescence assays, 6–8 seedlings from each of 3–4 independent lines carrying the *CCA1*:*LUCIFERASE* (*CCA1*:*LUC*) reporter (*pifQ CCA1:LUC* and wt *CCA1:*LUC) were grown for 8 days in 14 L:10 D. After spraying with 2.5mM luciferin (D-Luciferin, Potassium salt, Gold Biotechnology, St Louis, MO, USA) in 0.01% Triton X-100, the seedlings were transferred to a growth chamber mounted with a Hamamatsu ORCA II ER CCD camera (C4742-98 ERG; Hamamatsu Photonics, Hamamatsu City, Japan). Light was provided by red and blue light emitting diodes (LEDs), with regulated total fluence rates. Luciferase activity was imaged every two hours for at least four days. Images were analyzed with ImagePro software (Media Cybernetics, Inc., Bethesda, MD, USA). Data were imported into the Biological Rhythms Analysis Software System (BRASS; http://www.amillar.org) and analyzed with the FFT-NLLS (Fourier Transform-NonLinear Least Squares) suite of the program, as previously described [23]. Rhythms with a period between 15 and 35 h were taken to be within the circadian range. 

### 2.3. Leaf Movement Assays

Plants were grown on a Murashige and Skoog (MS) medium supplemented with or without 2% sucrose in 14 h:10 h, light:dark (100 µmol m^−2^ s^−1^) for eight days before being transferred to 24-well cell culture plates (Greiner Labortechnik, Kremsmünster, Oberösterreich, Austria), one plant per well. The plates were put into continuous light at 23 °C or 28 °C. The light sources are described in the figure legends for each experiment. Leaf movements were recorded every 20 min for 5–7 days by Panasonic CCTV cameras, model WV-BP120 (Matsushita Communications Industrial, Yokohama, Japan). Post-run analysis was performed using the ImagePro software. Traces of the Y-coordinate position of leaves were imported into BRASS software. Two to three replicates were carried out for each experiment. The results of the replicates were similar and so were averaged.

### 2.4. Sucrose Pulses

Sucrose pulse experiments were carried out essentially as described in [16]. *pifQ CCA1*:*LUC* plants and wt *CCA1*:*LUC* plants were grown for 10 days in 14 L:10 D 100 µmol m^−2^ s^−1^ provided by white LEDs on MS media without sucrose on 0.8 µm pore nylon mesh filters, in order to prevent the roots from penetrating into the media. The plants were transferred at dawn to 5 µmol m^−2^s^−1^ constant blue light (Bc), and luciferase activity was imaged at 1 h intervals. After 24 h in Bc, the membranes with plants were transferred onto MS media supplemented with 3% sucrose and irrigated with liquid MS + 3% sucrose for a total of 3 h. After the pulse, plants were washed with liquid MS media and transferred back onto MS plates for subsequent imaging. For the controls, the transfers were made using MS without sucrose. All the manipulations were done under green safe light. The time of the first peak after the sucrose pulse was determined using the BRASS “Peak time analysis” option.

### 2.5. RNA Isolation and Analysis

Ten to twelve seedlings were harvested per sample and total RNA extracted as previously described [24]. RNA samples were treated with DNase I (PerfeCTa DNAse from Quanta bio, Beverly, MA, USA) according to the manufacturer’s instructions. From each DNA-free RNA sample, 5 μL aliquots were used as a template to produce cDNA, using the qScript cDNA SuperMix (Quanta bio). 2.5 µL of template cDNA was used for quantitative RT–PCR reaction with SYBR green reagent (KAPA SYBR FAST qPCR kit Master Mix, Kapa Biosystems, MA, USA) according to the supplier’s protocol. Three technical repeats were made for each sample. Fluorescence was detected using the QuantStudio 12K Flex system (Thermo Fisher Scientific, MA, USA). PROTEIN PHOSPHATASE 2A (*PP2A*, AT1G13320), was used as a control for normalization [25]. Quantitation calculations were carried out using the 2–ΔΔCT formula as described by [26]. The primers are shown in Supplementary Table S1.

## 3. Results

### 3.1. Light-Dependent Roles for PHYTOCHROME INTERACTING FACTOR Regulation of Sucrose Signaling to the Oscillator

**Figure 1:**
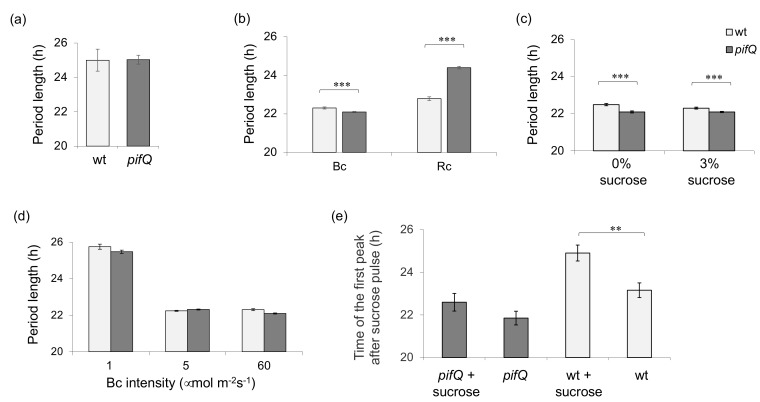
Blue and red light have different effects on PIF-mediated sucrose signaling. (**a**) The circadian period of leaf movements of wt and *pifQ* mutants grow on 2% sucrose containing medium for 8 days in 14 h: 10 h, light: dark (white light, 100 µmol m^−2^ s^−1^) before being transferred to constant blue light (Bc) of 60 µmol m^−2^ s^−1^. Average of 2 biological repeats, *n* ≥ 13. (**b**–**d**) Luciferase activity of *pifQ CCA1:LUC* and wt *CCA1:LUC* plants grown for 8 days in 14 h: 10 h, light: dark (white light, 100 µmol m^−2^ s^−1^) on medium (**b**,**d**) with or (**c**) with and without 3% sucrose and transferred to either (**b**) Constant blue light (Bc) (60 µmol m^−2^ s^−1^) and constant red light (Rc) (50 µmol m^−2^ s^−1^) or (**c**) Bc (60 µmol m^−2^ s^−1^) or (**d**) Bc of different light intensities (1, 5, 60 µmol m^−2^ s^−1^). (**e**) Sucrose pulse experiments were performed as described in Materials and Methods. ** *p* < 0.01 *** *p* < 0.001 (Student two-tailed *t*-test). Average of (**a**) 2 biological repeats. *n* ≥ 106, (**b**–**d**) 2–3 biological repeats. *n* ≥ 112 (**e**) 3 biological repeats *n* ≥ 36.

Our first goal was to determine whether and how blue light affected photosynthate regulation of the circadian oscillator. We grew *pifQ* and wt plants in Bc in the presence of 2% sucrose and examined the circadian rhythms of leaf movements. Figure 1a shows that, unlike under a wide range of fluences of constant red light (Rc) or in the dark (DD) [16], in Bc, the *pifQ* mutant did not show a long circadian period phenotype in the presence of sucrose. Expanding on this initial finding by analyzing LUC activity in *pifQ CCA1:LUC* and wt *CCA1:LUC* lines, we observed that, while *pifQ CCA1:LUC* plants grown on 3% sucrose in Rc had longer periods (*pifQ* 24.4 ± 0.07; wt 22.8 ± 0.1), plants growing in Bc had slightly but consistently shorter periods than wt *CCA1:LUC* (*pifQ* 22.1 ± 0.03; wt 22.3 ± 0.05; Figure 1b,c). Moreover, although increasing intensities of Bc caused a shorter period phenotype in both *pifQ CCA1:LUC* and wt *CCA1:LUC* lines in the presence of sucrose, there were only small differences between the responses of *pifQ CCA1:LUC* and wt *CCA1:LUC* (Figure 1d). 

Since under natural conditions both the levels of sucrose derived from photosynthesis and the plant’s sensitivity to photosynthates fluctuate during the course of the day, we examined the effects of treating *pifQ CCA1:LUC* and wt *CCA1:LUC* plants with a short pulse of sucrose in low Bc. Figure 1e shows that, by contrast, with the effect of sucrose pulses in Rc which caused a phase advance in wt plants [16], pulsing wt *CCA1:LUC* plants in Bc resulted in a phase delay, the first peak of *CCA1:LUC* occurred 24.9 h after the pulse, compared with 23.16 h for the non-pulsed control. However, like pulses in Rc, sucrose pulses in Bc did not significantly affect the circadian phase in *pifQ CCA1:LUC* plants under Bc (22.6 h after the pulse compared with 21.85 h for the non-pulsed control; *p* = 0.16 Student two-tailed *t*-test).

**Figure 2:**
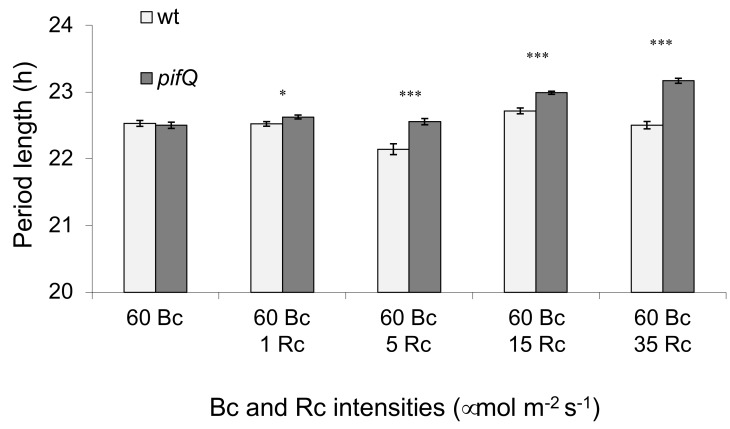
Low levels of red light are sufficient to restore the long period *pifQ* phenotype. *pifQ CCA1:LUC* and wt *CCA1:LUC* lines were entrained for 8 days in 14 h: 10 h, light: dark (white light, 100 µmol m^−2^ s^−1^) on medium, supplemented with 3% sucrose before being transferred to different constant light conditions (60 µmol m^−2^ s^−1^ Bc, 60 µmol m^−2^ s^−1^ Bc + 1 µmol m^−2^ s^−1^ Rc, 60 µmol m^−2^ s^−1^ Bc + 5 µmol m^−2^ s^−1^ Rc, 60 µmol m^−2^ s^−1^ Bc + 15 µmol m^−2^ s^−1^ Rc, 60 µmol m^−2^ s^−1^ Bc + 35 µmol m^−2^ s^−1^ Rc), and luciferase activity measured. Average of 2–4 biological repeats. n ≥ 103. * *p* < 0.05, *** *p* < 0.001 (Student two-tailed *t*-test).

**Figure 3:**
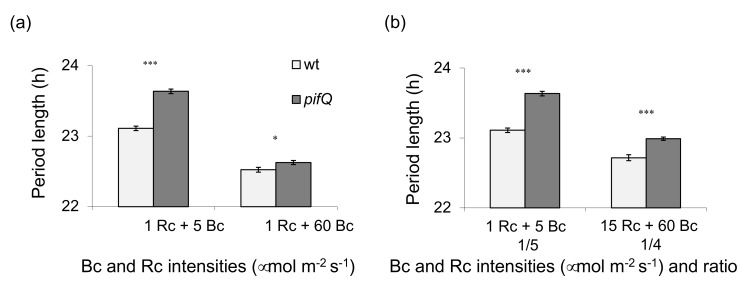
The red/blue light ratio is important for PIF regulation of the circadian oscillator. *pifQ CCA1:LUC* and wt *CCA1:LUC* plants were entrained for 8 days in 14 h: 10 h white light: dark (white light, 100 µmol m^−2^ s^−1^) on medium supplemented with 3% sucrose before being transferred to different constant light conditions and luciferase activity measured. (**a**) Similar Rc light intensity and varied Rc/Bc ratio. (**b**) Similar Rc/Bc ratios and varied total light intensity. Average of 4 biological repeats. n ≥ 213. * *p* < 0.05, *** *p* < 0.001 (Student two-tailed *t*-test).

The different effects of red and blue light on PIF-mediated sucrose regulation of the circadian oscillator (Figure 1) in [16] prompted us to examine how simultaneous irradiation with red and blue light affected sucrose signaling. Our results suggest that that even very low levels of red light (1 µmol m^−2^ s^−1^) partially reversed the blue light effects on the *pifQ* phenotype; with higher red-light intensities (more than 5 µmol m^−2^ s^−1^), *pifQ CCA1:LUC* plants showed a significantly longer period than wt *CCA1:LUC* (Figure 2). Moreover, under identical Rc conditions, the effect of pifQ on the period was affected by Bc levels; in 1 µmol m^−2^ s^−1^ red + 5 µmol m^−2^ s^−1^ blue (Rc/Bc is 1/5), *pifQ CCA1:LUC* plants had 0.52 h longer circadian periods than wt *CCA1:LUC*, but at Rc/Bc 1/60 (1 µmol m^−2^ s^−1^ red + 60 µmol m^−2^ s^−1^ blue), the difference in circadian period between wt *CCA1:LUC* and *pifQ CCA1:LUC* plants was much smaller (0.1 h, Figure 3a). To determine whether the pifQ phenotype was dependent on total light fluence or the Rc/Bc ratio, we examined plants under similar Rc/Bc ratios but with different intensities. Figure 3b shows that *pifQ CCA1:LUC* plants showed longer period phenotypes under both high and low total flux densities, suggesting that the ratio of Rc/Bc—rather than the total levels of Rc—may affect PIF-dependent entrainment of the oscillator in response to sucrose. This is consistent with the significant difference in period between wt *CCA1:LUC* and *pifQ CCA1:LUC* at similar (61 and 65 µmol m^−2^ s^−1^) fluences but different (Rc/Bc 1/60 and 1/12) Rc/Bc ratios (Figure 2). Together our results suggest that PIF mediation of photosynthate signaling to the circadian oscillator is light-quality-dependent. 

### 3.2. PHYTOCHROME INTERACTING FACTOR-Mediated Sucrose Signaling Is Temperature Dependent 

**Figure 4:**
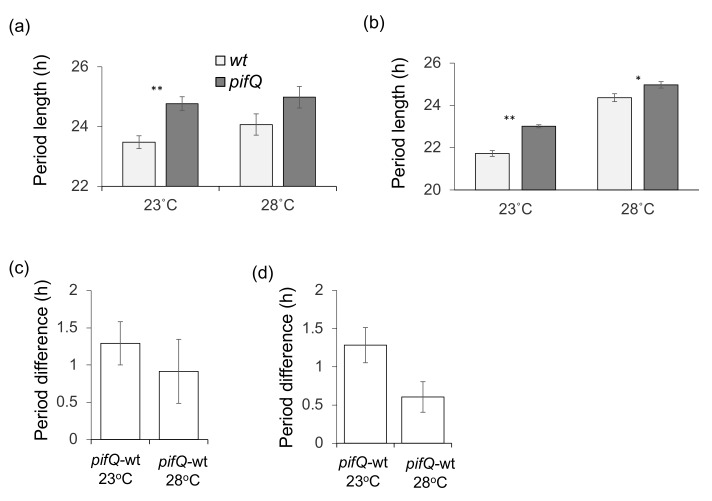
PIF-mediated sucrose signaling is temperature-sensitive. Circadian period of (**a**) leaf movements and (**b**) *CCA1:LUC* expression in wt plants and *pifQ* mutants, and differences in period length of plants at 23 °C and 28 °C, measured by (**c**) leaf movements or by (**d**) *CCA1:LUC* luminescence. Plants growing on (**a,c**) 2% sucrose or (**b**,**d**) 3% sucrose containing medium were entrained for 8 days at 23 °C before being transferred to LL provided by mixed LEDs (**a**,**c**) red (15 µmol m^−2^ s^−2^) green (10 µmol m^−2^ s^−2^) and blue (25 µmol m^−2^ s^−2^) or (**b**,**d**) red (50 µmolm^−2^ s^−2^) and blue (60 µmol m^−2^ s^−2^) at 23 °C or 28 °C. Average of (**a**) 3 biological repeats, n = 42–63, (**b**) 2–3 biological repeats, n = 107–174. * *p* < 0.05, ** *p* < 0.01 (Student two-tailed *t*-test). (**c**,**d**) Period difference (*pifQ*-wt) plotted with the standard error of the mean (SEM) (**c**) 23 °C *F* = 11.1, 28 °C *F* = 4.4 (**d**) 23 °C *F* = 30.3, 28 °C *F* = 8.94 (two-way ANOVA temperature, genotype factors).

PHYTOCHROME INTERACTING FACTOR 4 is known to participate in temperature sensing by plants [27], and the circadian oscillator has been shown to gate (i.e., restrict to certain times of day) PIF4-mediated thermo-responses [28]. We examined whether temperature affected the PIF-mediated sucrose entrainment of the circadian oscillator. Figure 4 shows that *pifQ* plants have a more reduced period phenotype of both leaf movements and CCA1:LUC at 28 °C than at 23 °C. *pifQ* mutants show a 1.3 h longer period of leaf movements than wt plants at 23 °C, compared with a 0.9 h longer period at 28 °C (Figure 4a,c). The difference in CCA1:LUC period between *pifQ* and wt plants is 1.3 h at 23 °C and 0.6 h at 28 °C (Figure 4b,d). Our results suggest that PIFs have more of a role in the sucrose entrainment of the oscillator at lower temperatures. 

**Figure 5:**
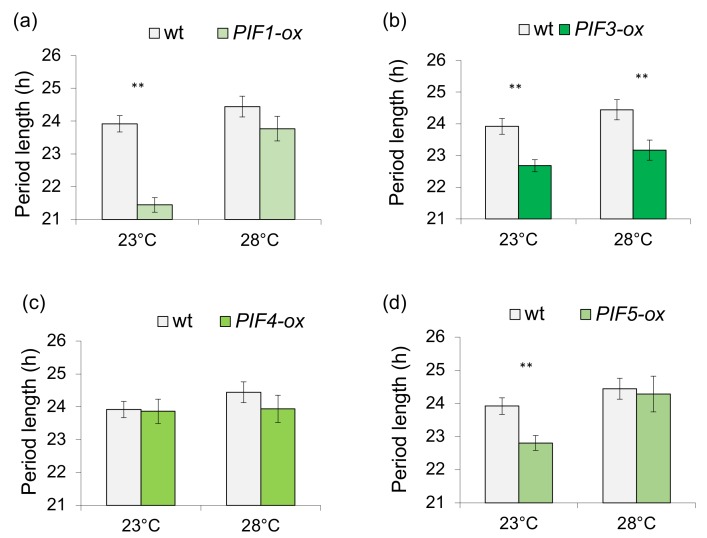
Circadian period of leaf movements of wt and *PIF*-overexpressing plants. Plants growing on 2% sucrose containing medium were entrained for 8 days at 14 h: 10 h, light: dark (100 µmolm^−2^s^−1^) at 23 °C before being transferred to constant white light (provided by fluorescent lamps; 50 μmol m^−2^ s^−1^; colour temperature 6500 K) at 23 °C or 28 °C. (**a**) *PIF1*-ox, (**b**) *PIF3-*ox, (**c**) *PIF4-*ox, (**d**) *PIF5-*ox. Average of 2–3 biological repeats, *n* = 17–41. ** *p* < 0.01 (Student two-tailed *t*-test).

Finally, to determine which PIF(s) participate in the temperature-dependent regulation of circadian rhythms, we analyzed individual *PIF*-overexpression lines (PIF-ox; Figure 5). In an RT–PCR analysis of *PIF* expression, each *PIF*-ox line showed that overexpression of individual *PIFs* did not have a significant effect on the levels of the other *PIF* genes examined (Supplementary Figure S1). Figure 5 shows that at 23 °C, all the *PIF*-ox lines, except *PIF4*-ox, showed a shorter period of leaf movements. PIF4 overexpression did not significantly affect the circadian period at either 23 °C or 28 °C (*p* = 0.89 and *p* = 0.34 for 23 °C and 28 °C, respectively by Student two-tailed *t*-test). By contrast, overexpression of PIFs 1 and 5 affected periods at 23 °C, but not at 28 °C (*p* = 0.79 and *p* = 0.18 for *PIF5*-ox and *PIF1*-ox, respectively by Student two-tailed *t*-test), while overexpression of *PIF3* caused a statistically significantly shorter period circadian phenotype at both, 23 °C and 28 °C. Thus, PIFs 1 and 5, but not PIF3, appear to have temperature-sensitive roles in the sucrose signal transduction to the circadian oscillator. 

## 4. Discussion

The results presented here show that, as in red light and in the dark, in blue light sucrose entrains the oscillator at dawn via PIF proteins. However, in contrast to red light, *pifQ* plants in blue light have a slightly but significantly shorter period (Figure 1). The levels of blue light required to inhibit sucrose-mediated entrainment are very low, comparable to those seen at the end of the night before sunrise [29]. Moreover, the red/blue ratio appears to affect PIF-mediated oscillator entrainment (Figure 2 and Figure 3), although we cannot rule out effects of total light fluence on period. A potential ecological significance of the red/blue regulated sensitivity to sugar availability might be to enable plants to distinguish between sucrose produced de novo from photosynthesis during the day when red light levels are high and the sucrose products of starch degradation at the end of the night when blue lights levels are higher [30]. Thus, the sucrose state of plant and light quality signals may interact to optimize the timing of plant processes to match their environments.

These findings also suggest that signaling pathways regulating sucrose activity in the circadian system in red and blue light may be interconnected. Phys and Crys provide the circadian oscillator with sensitivity to light. Both, Cry1 and PhyA mediate low-intensity blue light for period length control [31]. Moreover, Cry1 is necessary for PhyA signaling. PhyA is also active under low red light, while PhyB is active under high red light. PhyB does not participate in the mediation of blue light signal to the clock. Cry1 and Cry2 act redundantly in blue light perception and participate in blue and red light input to the oscillator [31,32]. The mechanism by which blue light blocks the PIF-regulated sucrose entrainment of the oscillator may involve an altered sensitivity of PIFs 4 and 5 to blue light, via their direct interaction with the blue light photoreceptors, Cry1, and Cry2 [33,34]. As PIF4 and PIF5 also act downstream of Phys [17,35], they may represent a molecular basis for the cross-talk between red/blue photosensory pathways. It is also possible that ZTL, FKF1, and LKP2, LOV-domain-based blue-light photoreceptors that regulate the circadian clock by controlling TOC1 and PRR5 stability [10,36,37,38], may be involved in the blue light regulation of PIF-mediated sucrose signal perception. 

A number of temperature-dependent roles for PIF activity have been previously reported [17]. At high temperatures, wt but not *pif4* plants show an altered morphology and reproductive development [27,39]. The expression of *PIF4* and *PIF5* is temperature regulated and is elevated in hypocotyls at high temperature [39]. PIF4 directly binds to the *FT* promoter in a temperature-dependent manner, leading to flowering induction at high temperatures [18]. PIF4 also regulates auxin biosynthesis and activity at high temperatures, resulting in increased levels of IAA in seedlings [40]. In the circadian system, temperature-dependent outputs of the oscillator are gated by PIF4 interactions with TOC1, resulting in the repression of warm-temperature activation of PIF4 target genes [28]. The functioning of the circadian system itself is, in turn, temperature-regulated. The expression of *CCA1*, *LHY*, *TOC1*, *GI* is affected by temperature conditions [41], and the temperature entrainment of the Arabidopsis circadian oscillator requires PRR3 and PRR7 [42]. During seed germination, sensitivity to daily temperature cycles is provided by PRR7 and TOC1, and the cyclic expression of *PRR7* and *TOC1* is stimulated by day/night temperature changes [43].

In this work we have shown that PIF-mediated sucrose signaling is also temperature-dependent (Figure 4 and Figure 5); there is a smaller difference between the period lengths of *pifQ* mutants and wt plants at higher temperatures (28 °C compared with 23 °C; Figure 4). *PIF1*-ox and *PIF5*-ox are temperature-sensitive; overexpression effects circadian period at 23 °C, but not at 28 °C. By contrast, *PIF3*-ox affects period phenotype in both high and low temperatures. Overexpression of *PIF4* did not significantly affect temperature sensitivity of the circadian period (Figure 5), possibly as a result of increased levels of *PIF4* in wt plants at high temperatures [44], resulting in wt and *PIF4*-ox plants having similar levels of *PIF4* under these conditions. Together with the discovery that *pif34*, but not *pif13* double mutants or *pif3* single mutants, have long period circadian phenotype [16,45], these data indicate that it is the presence and not the quantity of PIF4 that is most important for clock regulation.

In summary, the work described here demonstrates that the PIF-mediated entrainment of the Arabidopsis circadian oscillator by sugars is temperature- and light quality-dependent. PIFs act as a hub for environmental factors in the regulation of the clock. This finding gives us a deeper understanding of how the plants adapt their circadian systems to the environment, utilizing multiple signals—light, temperature and sucrose levels—to accurately determine the time of day. 

## Supplementary Materials

The following are available online at http://www.mdpi.com/2073-4425/9/12/628/s1, Figure S1: The effects of *PIF*-overexpression on levels of individual PIFs. Table S1: Primers, used for RT-PCR.
