# Supplementary material for: PIF-Mediated Sucrose Regulation of the Circadian Oscillator is Light Quality and Temperature Dependent
Source: Genes (Basel). 2018 Dec 13;9(12):628. doi: 10.3390/genes9120628 (PMC6316277; doi:10.3390/genes9120628)
Supplement: Supplementary file 1 [file genes-09-00628-s001.pdf]

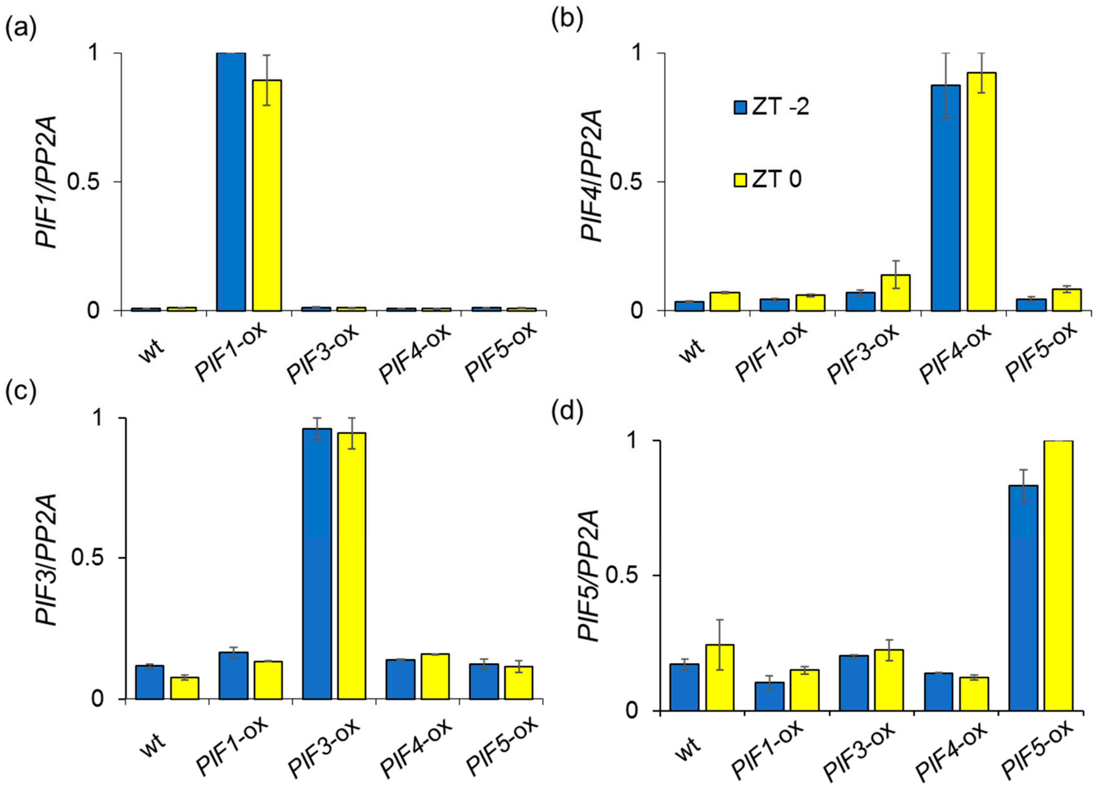

Figure S1. The effects of *PIF*-overexpression on levels of individual PIFs. PIF-overexpressing and wt plants were entrained for 8 days in 14 h: 10 h, light: dark, white light (100 µmol m^{-2} s^{-1}) before being transferred to LL. Samples were taken at ZT-2 and ZT0 (46 and 48 hours at LL, respectively). The average expression of **(a)** *PIF1*, **(b)** *PIF4*, **(c)** *PIF3* and **(d)** *PIF5* with SEM from two independent biological repeats is shown.

Supplementary table S1: Primers, used for RT-PCR.

PIF1	gccaccactactgatgaaactg	atgaacttcagcagcacgag
PIF3	gacggcgtgataggatcaac	catcgaagctttgtccacct
PIF4	aagtcgaaccaacgatcagg	ttgcaaagccttcattctctc
PIF5	cagaccagaagatgaattagt	acggttctctacgagcttgg
PP2A	aaacttgcgtgagggagaaa	ggaaaatcccacatgctgat
